# Modulation of the *Neisseria gonorrhoeae* drug efflux conduit MtrE

**DOI:** 10.1038/s41598-017-16995-x

**Published:** 2017-12-06

**Authors:** Giulia Tamburrino, Salomé Llabrés, Owen N. Vickery, Samantha J. Pitt, Ulrich Zachariae

**Affiliations:** 1 Computational Biology, School of Life Sciences, University of Dundee, Dundee, DD1 5EH UK; 2 Physics, School of Science and Engineering, University of Dundee, Dundee, DD1 4NH UK; 3 School of Medicine, University of St Andrews, St Andrews, KY16 9TF UK

## Abstract

Widespread antibiotic resistance, especially of Gram-negative bacteria, has become a severe concern for human health. Tripartite efflux pumps are one of the major contributors to resistance in Gram-negative pathogens, by efficiently expelling a broad spectrum of antibiotics from the organism. In *Neisseria gonorrhoeae*, one of the first bacteria for which pan-resistance has been reported, the most expressed efflux complex is MtrCDE. Here we present the electrophysiological characterisation of the outer membrane component MtrE and the membrane fusion protein MtrC, obtained by a combination of planar lipid bilayer recordings and *in silico* techniques. Our *in vitro* results show that MtrE can be regulated by periplasmic binding events and that the interaction between MtrE and MtrC is sufficient to stabilize this complex in an open state. In contrast to other efflux conduits, the open complex only displays a slight preference for cations. The maximum conductance we obtain in the *in vitro* recordings is comparable to that seen in our computational electrophysiology simulations conducted on the MtrE crystal structure, indicating that this state may reflect a physiologically relevant open conformation of MtrE. Our results suggest that the MtrC/E binding interface is an important modulator of MtrE function, which could potentially be targeted by new efflux inhibitors.

## Introduction

The introduction of antibiotics into clinical use against bacterial infections marked one of the most important milestones in medicine. However, the high evolutionary pressure caused by the widespread use of antimicrobial drugs has led to the rise of antibiotic-resistant bacterial strains^1^. In recent decades, antimicrobial resistance (AMR) has evolved into a major health problem, as many bacterial species have become insusceptible to a growing range of antibiotics, and we may soon face the prospect of a post-antibiotic era. Some bacteria, especially Gram-negative organisms including strains of *Neisseria gonorrhoeae*, have become pan-resistant, i.e. they can no longer be treated with any available antibiotic^2^. The exceptional urgency of addressing the emergence of bacterial multi-drug and pan-resistance has therefore been widely recognised by national and international health authorities^3^ and *N. gonorrhoeae* has been named amongst the 12 bacteria prioritised by the WHO for accelerated research efforts to develop new antibiotics^4^.

Gram-negative bacteria possess a double-membrane cell envelope, which acts as a highly efficient barrier for the inward permeation of drugs. Antibiotic agents enter these organisms predominantly via porin channels in the outer membrane, and are often expelled directly from the periplasm, located between the two bilayers, by active drug efflux pumps. Many highly resistant forms of Gram-negative bacteria display a combination of single-point mutations in porin channels and upregulation of efflux pump expression^5,6^. Amongst these, the major drivers of super-resistant phenotypes in Gram-negative bacteria are tripartite efflux pumps, protein complexes which span both the inner and outer membrane and form a continuous aqueous pathway from the inner membrane to the external medium^7^. The most clinically relevant family of Gram-negative efflux pumps is the resistance-nodulation-cell division (RND) superfamily, consisting of three major elements: an inner membrane pump protein (IMP), an outer membrane channel protein (OMP), and a membrane fusion protein (MFP) connecting the aforementioned components^8^. The importance of active efflux for the development of bacterial resistance has been impressively demonstrated in experiments, showing that multidrug-resistance can be reversed by knocking out the expression or inhibiting the function of efflux pumps^9,10^. It is therefore important to illuminate the mechanisms underpinning rapid drug expulsion. For example, targeting this major driver of multidrug resistance by new inhibitors may reduce the efficiency of efflux pumps and thereby restore the susceptibility of resistant bacteria to existing antibiotics. In addition, the modulation of efflux pumps may narrow the compound spectrum of expelled drugs and enhance the uptake of therapeutics into Gram-negative bacteria.

**Figure 1:**
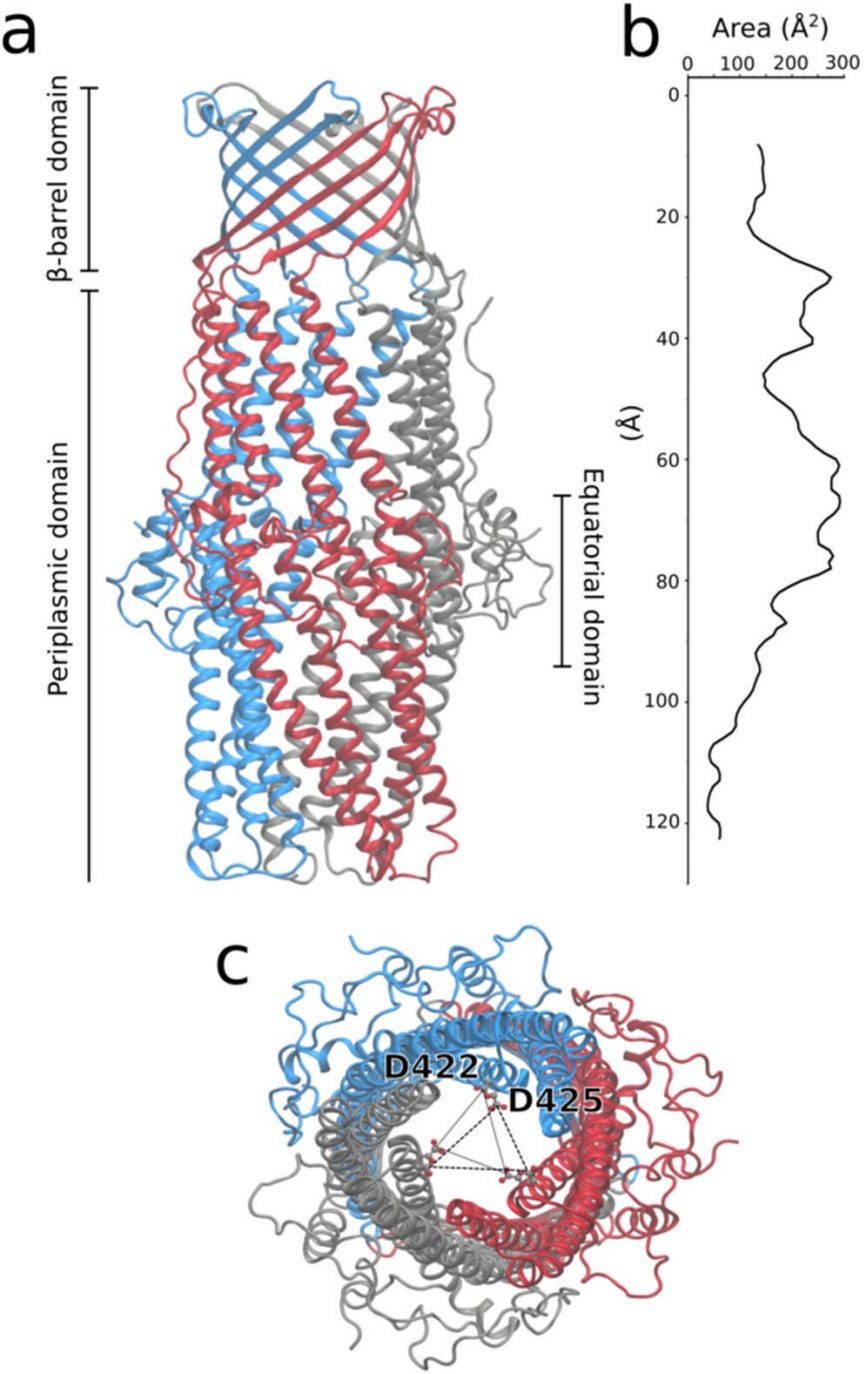
(**a**) Overall structure of the trimeric MtrE efflux protein channel (PDB code: 4MT0). Each subunit consists of a *β*-barrel domain, embedded in the outer membrane, an *α*-barrel domain, spanning ~ 100 Å inside the periplasm and an equatorial domain, mostly unstructured, located in the middle of the *α*-barrel. (**b**) The reported crystal structure of MtrE corresponds to a conduit with a comparably large cross-sectional area, in which the major constriction site is located at the tip of the periplasmic domain (110–120 Å from the extracellular exit along the pore axis). (**c**) The narrowest site in the channel is formed by two concentric aspartate rings comprising three D422 and D425 in the trimer (conserved across the other characterized OMPs), which are thought to act as a selectivity gate. Viewed from the periplasmic entrance, the ring formed by D422 is more proximal, the one from D425 more distal.

In the case of *N. gonorrhoeae*, targeting efflux is of particular urgency as some of its strains have developed pan-resistance, making efficient medical treatment by antibiotics impossible^11^. The most highly expressed tripartite efflux pump in *N. gonorrhoeae* is MtrCDE^12^. Its outer membrane channel component, MtrE, has recently been structurally characterised (PDB code: 4MT0^13^). As in other RND homologues, MtrE is a homotrimeric protein consisting of three domains: a *β*-barrel domain embedded in the outer membrane, an *α*-barrel domain, projecting over 100 Å into the periplasm, and an equatorial, mostly unstructured domain located in the middle of the *α*-barrel (Fig. 1a). Its putative gating region is located at the periplasmic tip and is formed by two concentric rings of conserved aspartate residues (D422 and D425; Fig. 1b,c). So far, MtrE is the only structurally determined wild type RND-OMP showing an open conformation in this region, expected to maximise efflux through the duct.

Here, we present planar lipid bilayer recordings and all-atom molecular dynamics simulations characterising the conductance of the MtrE RND exit tunnel. Our results show that binding of the membrane fusion protein MtrC to the external face of MtrE stabilises the open state of the channel. Our findings thus highlight the contact region between the OMP and the MFP of MtrCDE as a switch between open and closed states of the outer drug efflux conduit. This interface may therefore represent an attractive site for potential molecular intervention.

## Results

### Planar Lipid Bilayer Experiments

First we performed electrophysiological measurements, in which MtrE was embedded into symmetric POPE planar lipid bilayers. Planar lipid bilayer electrophysiology has frequently been used to investigate the conductance and gating of OMPs in response to membrane voltages^14–17^. The method makes use of simplified membrane models, as opposed to the highly complex architecture of bacterial outer membranes, which consists of asymmetric lipid bilayers, with inner leaflets containing phospholipids and outer leaflets composed mainly of lipopolysaccharides^18^. It has however been shown that electrophysiology in these simplified membrane models is a valid approach to characterise the translocation of ions across outer membrane channels^19^. Furthermore, the gating region of efflux outward gates is located at a large distance from the membrane in the periplasmic space, and therefore it is expected that the simplified membrane does not substantially affect results on the gating of these outer conduits^16^.

It has further been demonstrated that the direction of insertion of OMPs into membranes is entirely determined by the protein structures^20,21^, and that OMPs spontaneously insert unidirectionally in planar lipid bilayers^16^. In addition, MtrE possesses a large polar periplasmic domain, which is unlikely to traverse the membrane (Fig. 1a). Since we have exclusively added the OMP to the membrane face corresponding to the periplasmic side (cis-chamber; n = 10), it is highly probable that MtrE is unidirectionally inserted into the bilayer in our experiments, despite the symmetry of the POPE leaflets.

**Figure 2:**
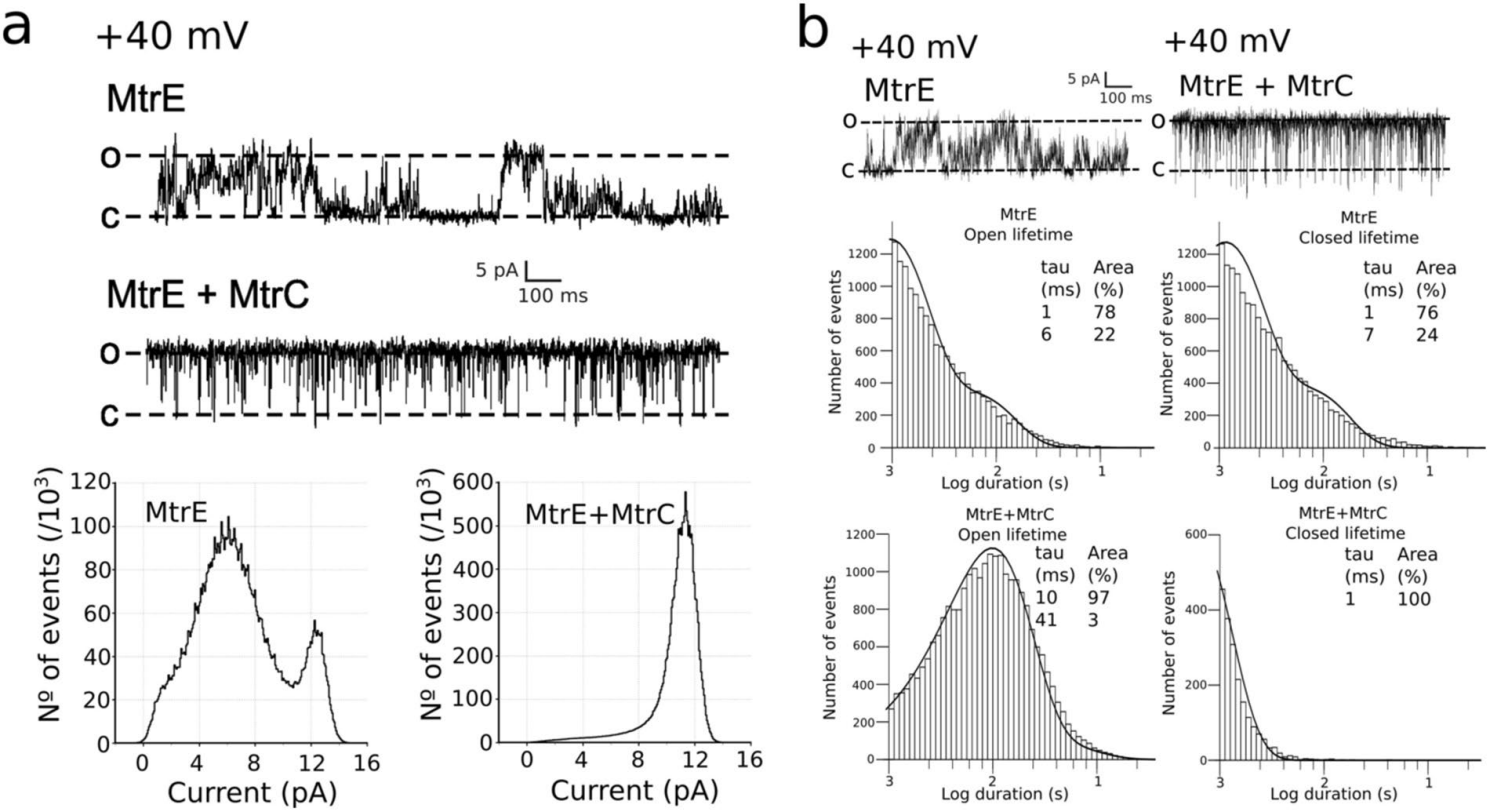
(**a**) Single channel experiments reveal that MtrE can dwell between 3 different semi-open states. The addition of the adapter protein MtrC stabilizes the most open conformation. (**b**) Lifetime analysis uncovers that the effect of MtrC is to significantly reduce the closed lifetime of MtrE, so that the channel resides for shorter intervals in the closed state.

When voltage-clamped at + 40 mV, the fully open state of MtrE was characterised by a unitary current amplitude of 11.5 pA (Fig. 2a). MtrE was also found to open to multiple sub-conductance open states that were approximately 18% (2 pA) and 60% (7 pA) of the fully open state (Fig. 2a). Addition of the binding partner MtrC to the *cis*-chamber significantly increased the open probability of MtrE from 0.35 ± 0.11 to 0.86 ± 0.12 (n = 3) and caused MtrE to gate predominantly to the fully open state (Fig. 2a). Transitions to the sub-conductance open states of MtrE following the addition of MtrC were no longer resolved.

**Figure 3:**
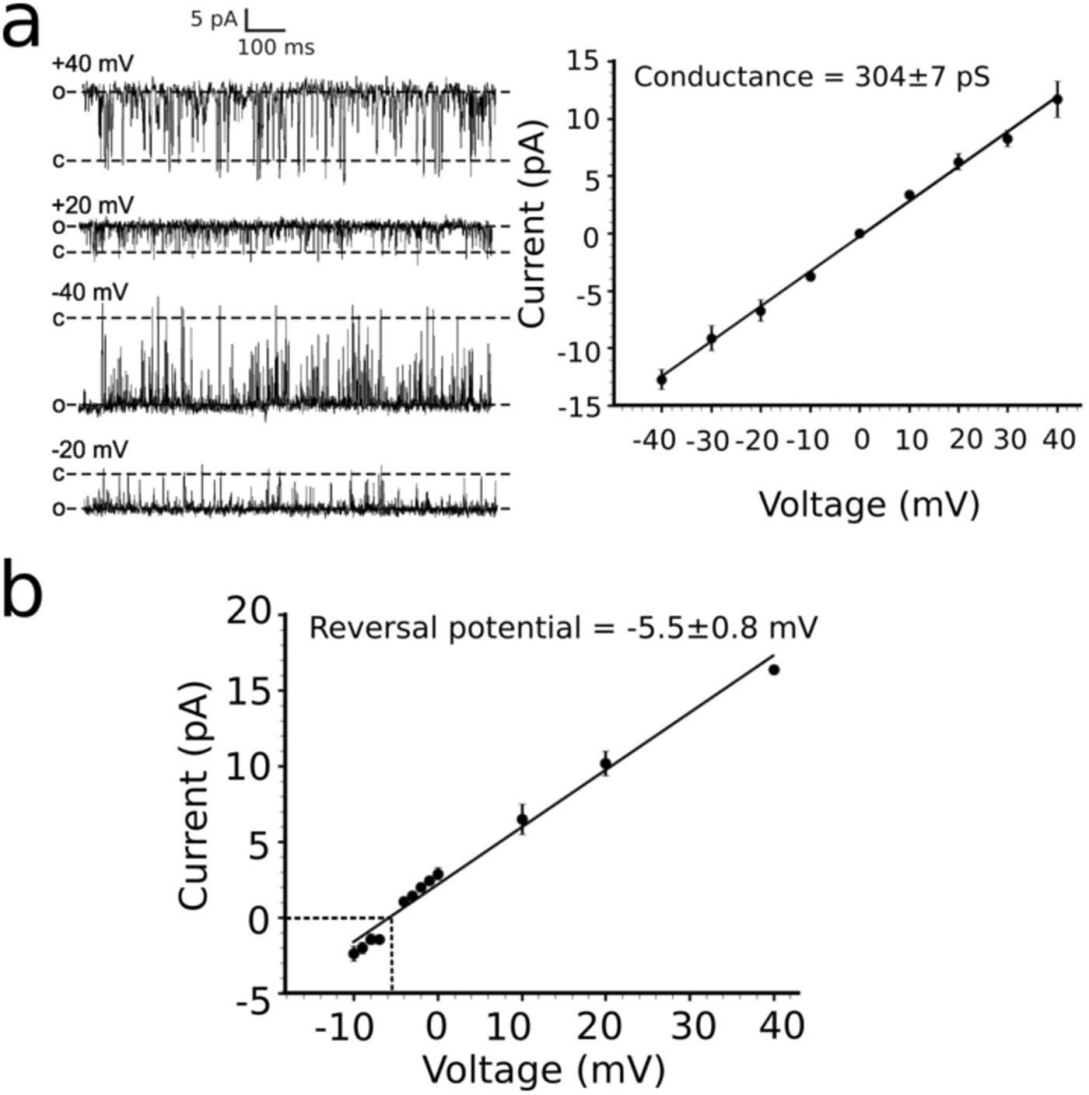
(**a**) The I/V plot of the MtrC/E protein complex is linear in the experimental voltage range (between −40 and +40 mV) and the conductance for the fully open state is 304 ± 7 pS. (**b**) The reversal potential of MtrE in non-symmetrical conditions (*trans* 210 mM KCl:*cis* 460 mM KCl), indicated by a dotted line, is at −5.5 ± 0.8 mV revealing slight cation selectivity.

When only a single MtrE channel was gating in the bilayer, lifetime analysis revealed that in the absence of the MtrC membrane fusion protein MtrE displayed fast flickery gating (Fig. 2b). The addition of MtrC to the periplasmic face of MtrE altered channel gating causing the channel to dwell for longer sojourns in the fully open state, demonstrated by an increase in the apparent open time from 1 ms to 10.5 ms (Fig. 2b). This suggests that MtrC alters the conformation of the MtrE protein and stabilizes the channel in the fully open state. In 3 out of 13 experiments, MtrE gated predominantly to the fully open state even in the absence of MtrC adapter protein, suggesting that once in this state, channel openings are stabilised. Construction of a current-voltage relationship for the fully open state of MtrE displayed an open channel conductance of 304 ± 7 pS (Fig. 3a).

Under non-symmetrical conditions (*trans* 210 mM KCl:*cis* 460 mM KCl), the reversal potential shifted to −5.5 ± 0.8 mV (Fig. 3b), revealing that MtrE is approximately 1.7 fold more permeable for K^+^ than for Cl^−^. This characterises MtrE as a slightly cation selective channel. The best-studied OMP homologues, TolC from *E. coli* and OprM from *P. aeruginosa*, also display a preference for cations, however to a much larger degree. For instance, TolC has been found to be 16.5 fold more permeable for K^+^ than for Cl^−^^22^, and the selectivity of OprM has been estimated to be similar to that of TolC^23^.

### MtrE Dynamics and Computational Electrophysiology

Previous experiments at the cellular level have shown that substrates cannot traverse MtrE from the extracellular space to the periplasm when complex formation to the tripartite pump is inhibited^24^. This finding suggests that MtrE requires the interaction with other pump components to induce the fully open conformation, which is expected to maximise the efficiency of efflux. In the crystal structure, the Asp gating rings (Fig. 1c) and the overall conformation of MtrE reflect an open state, wide enough to accommodate even larger substrate molecules (Fig. 1b), although its complex binding partners are absent. We were therefore interested if the crystallographically observed conformation corresponds to the fully open state of MtrE.

The majority of our simulations of MtrE immersed in POPC and POPE membranes show rapid closing of the periplasmic gating region. Despite displaying complex dynamics, especially in the loop region and the outer helices, the inner helices rapidly adopt a continuously closed state on the time-scale of most of our simulations. This finding indicated that the protonation state of the Asp gating rings may act as a potential modulator of the closing transition^25^. We therefore tested if a different protonation state of the Asp side chains affected channel closing. As shown in Supplementary Fig. S1, protonation of the Asp gating rings slightly lowers the propensity for closing, however a consistently stable open state of the gating region is still not observed. This observation suggests that additional factors are likely to play a role in stabilising the open state of the channel. Our experimental data show that binding of MtrC leads to the stabilisation of MtrE in an open conformation for substantial lengths of time. Taking these results together, we suggest that binding partners are required to retain MtrE in a fully open conformation, while protonation changes within the gating region, possibly induced by binding, may further stabilise the open state. In addition, our results indicate that crystal contacts may be able to at least partially compensate for the lack of interactions with other pump components to retain MtrE in the open state. In our single channel experiments, we observed MtrE to adopt a mostly closed state in the absence of MtrC, while the pH of the solution was not altered, emphasising the major role of the MtrE/MtrC contacts which we find for gating the channel.

**Figure 4:**
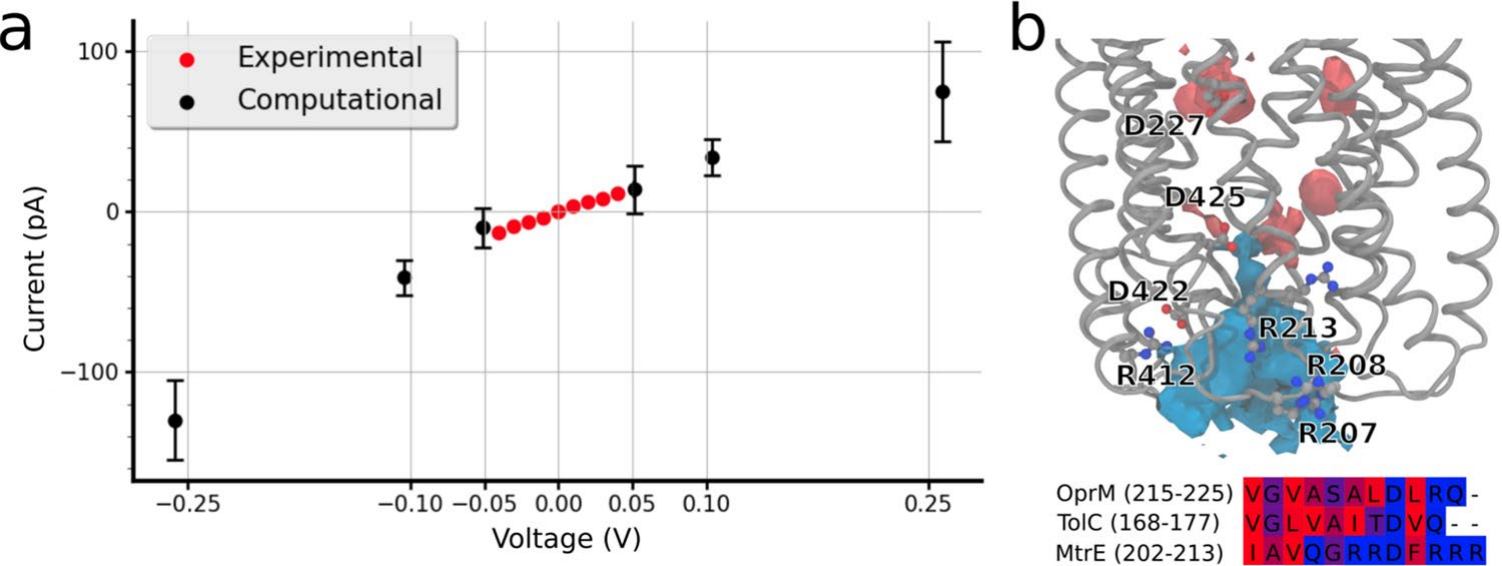
(**a**) Red dots display the experimental current-voltage relationship in symmetrical 210 mM KCl solution, black dots show the current/voltage relationship obtained from the MD simulations near the experimental voltage range. Voltages of ±0.05, ±0.10 and ±0.25 V were applied along the z-axis using external electric fields as implemented in GROMACS^57^, together with the charmm36 force field and positional restraints of 200 kJ/mol · nm^2^ on MtrE heavy atoms as detailed in the text. We observe excellent agreement between the experimental and the computational currents. The computational x-axis and the experimental x and y-axis error bars are smaller than the data points. (**b**, bottom) Sequence alignment of the three electrophysiologically characterised efflux OMPs. MtrE shows an abundance of arginine residues in the periplasmic loop region in addition to a series of aspartate residues lining the interior of the pore, which is not present in homologous proteins. (**b**, top) The density map of K^+^ (contoured at 2% SD, in red) and Cl^−^ (contoured at 5% SD, in blue) highlights the influence of this highly basic region on ion flow through the channel.

To date, there is no experimental information available on the three-dimensional structure of MtrC, nor its mode and position of binding to MtrE. To mimic the stabilising effect of the membrane fusion partner, we therefore restrained the overall backbone conformation of the protein and investigated by computational electrophysiology simulations (CompEL) if the pore geometry captured in the crystal can explain the experimentally observed channel currents. Due to the limited time-scale of the simulations, we used slightly raised transmembrane voltages compared to our experimental conditions, in order to drive ion permeation and ensure sufficient sampling^26^. Figure 4 shows that the current we obtain for single open MtrE conduits is generally in good agreement with our experimental data. The estimated maximum conductance of the open pore is 324 ± 34 pS, which is similar to the maximum experimental conductance of 304 ± 7 pS. In the simulations, we observe ion selectivity ratios between 1:1.2 ± 0.5 (K^+^:Cl^−^, at 100 mV) and 1:1.3 ± 0.5 (at −100 mV). These voltages were chosen as they are close to the experimental range of voltages, while at the same time allowing us to record sufficient sampling of ion permeation for robust selectivity estimates. The selectivity values are in generally good agreement with the experimental ion selectivity, although Cl^−^ ions show a slightly higher permeability in our simulations compared to the experiments.

We modelled an additional MtrE conformation, based on the most dilated state observed for TolC so far, where it is in complex with the complete RND machinery MacAB-TolC^27^ (Supplementary Fig. S2). For this model, we measure an *in silico* conductance of 597 ± 70 pS, which is considerably higher than our experimental conductance. Our data therefore suggests that the crystal structure of MtrE is a good representation of the overall conformation of the fully open state of this conduit. Our results further show that protein-protein contacts between MtrE and MtrC stabilise the fully open pore in the electrophysiological experiments, whereas in isolation, such as in our computer simulations, the open conformation of MtrE is likely to undergo rapid transitions to more closed states unless restrained. Supporting this notion further, we constructed a molecular model of the bound state of MtrE and MtrC, based on the homologous complex of AcrAB-TolC resolved by electron cryo-microscopy, which exhibits a similar degree of opening of the OMP gating region as the MtrE crystal structure^8^. Simulations of this complexed model indicate an increased tendency to remain in an open state without undergoing further protonation changes (Fig. S3).

**Figure 5:**
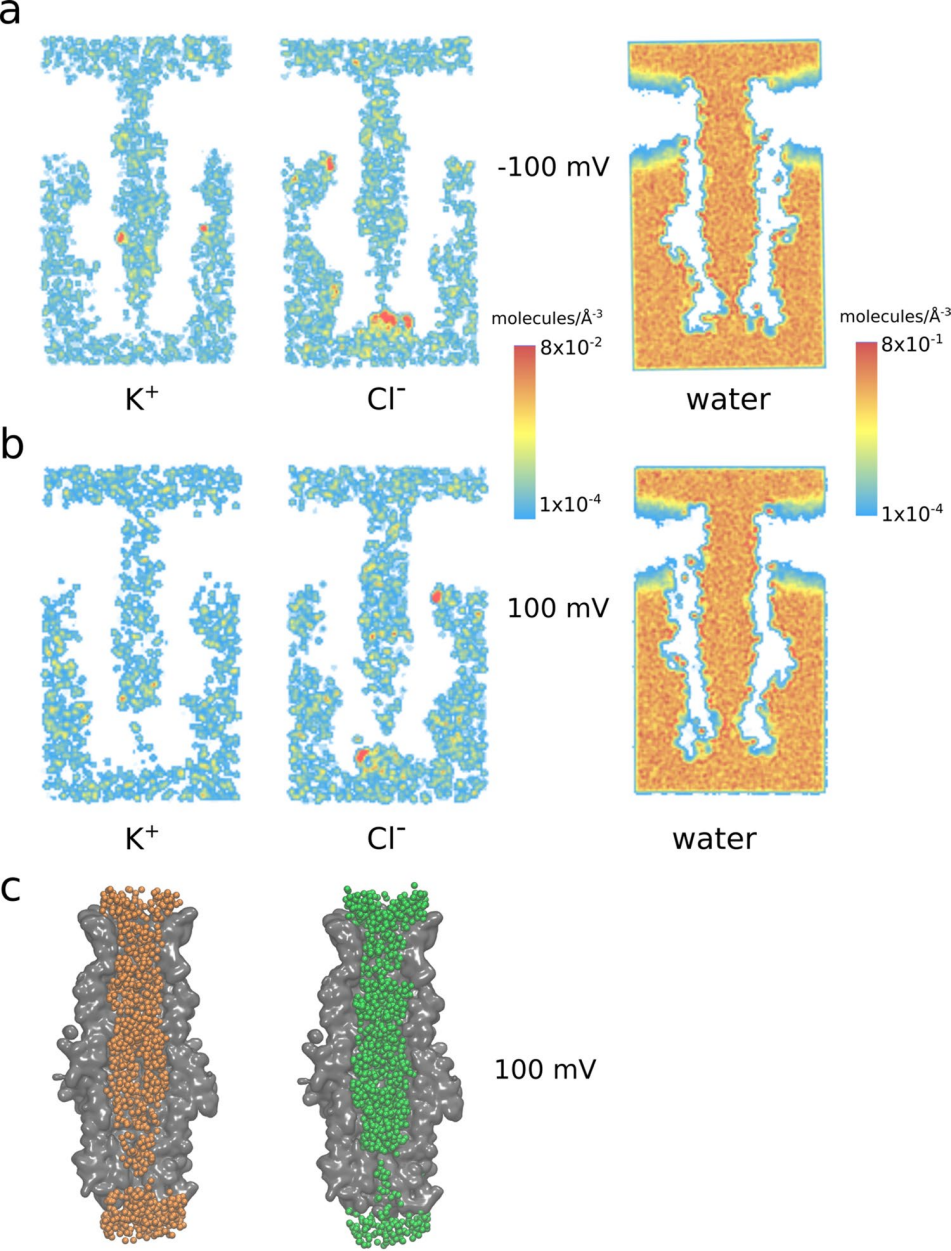
(**a**,**b**) Pathways of K^+^ and Cl^−^ ions in the MtrE channel (depicted as ion density) and average water density in the channel during simulations at (**a**) +100 mV and (**b**) −100 mV. The panels show the extracellular side of MtrE at the top and the periplasmic gate at the bottom of each figure. Under both conditions, a thinning out of the ion density at the periplasmic gate can be observed, especially for K^+^ ions. (**c**) Superposition of all K^+^ (orange) and Cl^−^ (green) ions during 250 ns (1 frame every 2.5 ns, 100 total frames) at +100 mV clearly shows this constriction of the ion pathways within the gating region, representing the major bottleneck for ion permeation.

Our analysis of ion trajectories in the simulations of MtrE under voltage (Fig. 5) shows that, although both anions and cations generally occupy most parts of the MtrE channel lumen to similar extent, there are regions in which important differences are observed. Especially the periplasmic gating region, but also sections of the transmembrane *β*-barrel show a substantially reduced K^+^ density, particularly evident at negative membrane potentials (Fig. 5a,b, left). A superposition of the positions of ions along the trajectories shows the constrictions observed for the cation pathway at the periplasmic entrance, highlighting the importance of this gating region, which includes a high density of arginine residues (see Fig. 4), for the control of ion conduction. Furthermore, we observe that the channel lumen and gating region are well-hydrated in the open state of MtrE (Fig. 5a,b, right).

Dewetting transitions play a major role in the gating process of many membrane channels^28,29^. To ascertain if dewetting plays a part in the closing of MtrE, we further analysed the simulations of MtrE, in which the channel rapidly adopted a closed state (Fig. S1). We found that even when the gating region shows a minimal level of openness, occupation with water and a continuous water chain between the extracellular and periplasmic side are still observed (Fig. S1B). Our findings suggest that dewetting of MtrE is unlikely to underpin the gating of this channel for ion conduction.

## Discussion

Both our experimental data, obtained from single-channel electrophysiology, and our computational results suggest that the channel MtrE is mainly present in a closed state when it is unbound from the rest of the efflux machinery. These findings are in agreement with electrophysiological recordings on homologous OMPs^16,30^, and biochemical data on MtrE^31,32^. Our results show that the interaction of the adapter protein MtrC with MtrE is sufficient to stabilise the complex in an open state, which conducts large ion currents in accordance with the cross-section of the channel observed in the MtrE crystal structure^13^. This is, to our knowledge, the first time that the fully open state of an OMP from an efflux pump could be characterised without inserting mutations in the gating region, as previously reported, e.g., for TolC from *E. coli* ^30^.

We attribute the subconductance states we observe in the electrophysiological recordings to the range of closed conformations we find in the MD simulations, which show a remaining open channel cross-sectional area of about 30–40 Å^2^ and continuous hydration of the pore (Fig. S1). As opposed to TolC, which shows a strong preference for cations^22^, the cation selectivity we observe for MtrE is about 10-fold smaller. In the simulations, a region of diminished cation density in the channel coincides with an abundance of arginine residues in the loop region at the periplasmic tip of MtrE, which precedes the channel-internal conserved aspartate gating rings from the periplasmic side, suggesting a role for these arginine groups in determining the ion selectivity of MtrE (Figs 4 and 5). Ultimately however, additional experiments will be required to unequivocally identify the determinants of the lowered level of MtrE ion selectivity. Notably, the major outer membrane porins of *Neisseria*, PorA and PorB, and of related organisms (e.g. Omp32 from *Delftia acidovorans*), show strong anion selectivity^33–35^ in contrast to the cation-selective porins of other Gram-negative organisms (such as OmpF from *E. coli*)^36^. The reduced cation-selectivity of *N. gonorrhoeae* MtrE could thus be linked to an increased inward permeability of the outer membrane of *Neisseria* species for anions compared to other Gram-negative bacteria.

All of these previous findings, together with our results, suggest that the inward and outward permeability of neisserial outer membranes differs substantially from that of model organisms, frequently used to investigate the determinants of Gram-negative cell wall permeation, such as *E. coli*. In particular, the uptake and efflux of antibiotics may be underpinned by different principles. According to our data, the effect of different model lipids on MtrE function is rather small, but the complex membrane composition of neisserial outer membranes may add a further layer of modulation and control of neisserial OMPs, which may differ from observations made in other Gram-negative bacteria.

The differences to permeation across *E. coli* TolC, for example, may have important consequences for the design of antibacterials against these difficult-to-treat Gram-negative pathogens. In particular, *N. gonorrhoeae* strains are amongst the most antibiotic-resistant bacteria, displaying pan-resistance against all presently available antibiotic agents^4,11^. This confers a high degree of urgency to the development of novel therapies against *N. gonorrhoeae* infections.

Active drug efflux of a broad spectrum of antimicrobials is one of the major factors driving the development of resistance in Gram-negative bacteria^2^. Our results suggest that the less stringent selectivity for cations in MtrE we found here may contribute to the efficiency with which a very wide range of antibiotics is expelled from *N. gonorrhoeae* by the MtrCDE efflux system. The diminished cation-selectivity may however also allow the exploration of new chemical space for the design of clinically usable efflux inhibitors. Most previous attempts at designing efflux pump inhibitors have failed in clinical studies due to toxicity problems related to the cationic pharmacophore, which is required for efficient competitive inhibition of the pumps^37^. Some inhibitors were also targeted against the internal Asp gating rings of the OMP, but clinical success was not achieved by these cationic inhibitors either^38^. The different electrostatics of the MtrE interior, and its reduced preference for cations, may enable drug researchers to design novel inhibitors with different charge properties.

Importantly however, our results indicate that it may not be necessary to block the outer gate of the efflux system through orthosterically binding inhibitors. We show that the opening of MtrE is regulated by allosteric binding events of the adapter protein MtrC on the periplasmic outer face of the pore. Association with MtrC alone is sufficient to keep MtrE in a prolonged open state. The binding of MtrC may be linked to further protonation changes at the interface and pore interior of MtrE. These findings suggest that the MtrE-MtrC binding interface may be an attractive targeting site for the development of allosterically acting efflux inhibitors. These inhibitors would no longer compete for the orthosteric drug binding sites in the efflux pumps, but still regulate the openness of, and thereby the efficiency of drug expulsion across, the outward conduit. This new modulation mechanism may potentially facilitate the design of a new type of inhibitor, avoiding previous chemotypes known for their toxicity.

## Methods

### Computational methods

The 3.29-Å resolution crystal structure of MtrE (PDB code: 4MT0)^13^ was used as a starting structure. The sulfate ion was removed and the N- and C-terminal residues were capped using acetyl and N-methyl amide groups, respectively. We note that there is relatively weak electron density for residues 203–212 in the crystal structure. All atomistic molecular dynamics simulations were performed using the GROMACS-5.1.1 software package^39^. We tested the robustness of our results by using different forcefields.

First, the protein was embedded in pre-equilibrated and solvated POPC (1-palmitoyl 2-oleoyl sn-glycero 3-phosphatidyl choline) bilayers containing 288 lipid molecules (177 after insertion of the protein) using the GROMACS g_membed utility^40,41^. The system was solvated and K^+^ and Cl^−^ ions were added to neutralise the system and to reach a concentration of 200 mM. Here, the Amber ff99SB-ILDN force field was used for the protein^42,43^, and Berger parameters adapted for use within the Amber ff99SB-ILDN force field were employed for the lipids^44,45^. The SPC/E model was used for the waters^46^ and Joung/Cheatham III parameters were employed for the ions^47^. The systems were minimised and then equilibrated with position restraints on protein heavy atoms of 1000 kJ/mol · nm^2^ for 20 ns. Water bond angles and distances were constrained by SETTLE^48^, while all other bonds were constrained using the LINCS method^49^. The temperature was kept constant at 310 K, using the v-rescale method^50^ with a time constant of 0.2 ps. The pressure was kept constant throughout the simulations at 1 bar, using a Berendsen barostat^51^ with semi-isotropic coupling. The application of the virtual site model for hydrogen atoms^52^ allowed the use of a 5-fs time step during the simulations. For the CompEL simulations, the protocol described by Kutzner *et al*.^26^ was used; the system was duplicated along the z axis to construct a double bilayer system, and ionic imbalances from 2 to 6 Cl^−^ ions were used between the aqueous compartments to generate a range of transmembrane potentials from ~−500 to ~500 mV. Some simulations made use of protein backbone heavy atom position restraints of 200 kJ/mol · nm^2^ or 1000 kJ/mol · nm^2^ as indicated in the text.

Furthermore, in additional sets of simulations, we employed the CHARMM36 force field for the protein, lipids and ions^53^. For water molecules, the TIP3 model was used^54^. The temperature was kept constant at 310 K, using the Nose-Hoover method^55^ with a time constant of 0.1 ps. The pressure was kept constant throughout the simulations at 1 bar, using a Parrinello-Rahman barostat^56^ with semi-isotropic coupling. Here, a 2-fs time step was used, and the proteins were inserted in a POPE (1-palmitoyl 2-oleoyl sn-glycero 3-phosphatidyl ethanolamine) membrane. A constant electric field was applied to generate membrane potentials from ~−250 to ~250 mV^57^. The systems were constructed by using the CHARMM-GUI webserver^58^. In the production simulations, protein backbone heavy atom position restraints of 200 kJ/mol · nm^2^ were applied.

Structural alignment was performed using the Jalview suite^59^, together with the Clustal W program^60^. Homology modelling was performed with the MODELLER9.16 suite^61,62^, using the dilated Cryo-EM structure of TolC^27^ (Fig. S2). During the modelling process, symmetry restraints were applied on MtrE Cα atoms, to take into account the threefold symmetry of the protein.

### Protein production

The MtrE protein was produced based on a previous protocol by Lei *et al*.^13^, but using the different expression plasmid pET28a. It was solubilised from the membrane in n-dodecyl-*β*-D-maltoside (DDM). The MtrC protein was produced based on a previous protocol by Janganan *et al*.^32^, increasing the growth period to 18 hours to yield a sufficient amount of protein. Analytical gel filtration suggested that the protein runs in several multimers, mainly dimers and hexamers, which correlates well with the protocol used^32^. The purity of the samples was confirmed by SDS-PAGE, and their identity was confirmed through peptide mass fingerprinting (Fig. S7), conducted by the FingerPrints Proteomics Facility of the University of Dundee.

### Conductance measurements and analysis

Current recordings were monitored under voltage-clamp conditions using a BC-525C amplifier (Warner Instruments, Harvard) following established methods^14,63^. Recordings were low-pass filtered at 10 kHz with a 4-pole Bessel filter, digitized at 100 kHz using a NIDAQ-MX acquisition interface (National Instruments, Texas, USA). Data were recorded to a computer hard drive using WinEDR 3.6.4 (Strathclyde University, Glasgow, UK). The recordings were subsequently filtered at 800 Hz(−3 dB) using a low-pass digital filter implemented in WinEDR 3.6.4. Experiments were performed at room temperature (20–22 °C). The MtrE protein was added to the *cis* chamber and incorporations made at +40 mV, under continuous stirring. Unless otherwise stated, single-channel events were recorded in symmetrical 210 mM KCl. For selectivity measurements, the *cis*-chamber contained 510 mM KCl and the *trans*-chamber contained 210 mM KCl. Selectivity was computed using the Goldman-Hodgkin-Katz equation. E_rev_ was taken as the voltage at which no current flow was detected and was corrected for junction potential. Lifetime analysis was performed using TAC and TAC-fit software (Bruxton, Seattle, WA).

## Electronic supplementary material

Supplementary Information
