# Supplementary material for: Modulation of the Neisseria gonorrhoeae drug efflux conduit MtrE
Source: Sci Rep. 2017 Dec 6;7:17091. doi: 10.1038/s41598-017-16995-x (PMC5719041; doi:10.1038/s41598-017-16995-x)
Supplement: Supplementary file 1 — Supplementary Information [file 41598_2017_16995_MOESM1_ESM.pdf]

Modulation of the Neisseria gonorrhoeae drug efflux conduit MtrE

Giulia Tamburrino, Salomé Llabrés, Owen N. Vickery, Samantha J. Pitt, Ulrich Zachariae

Supplementary Material

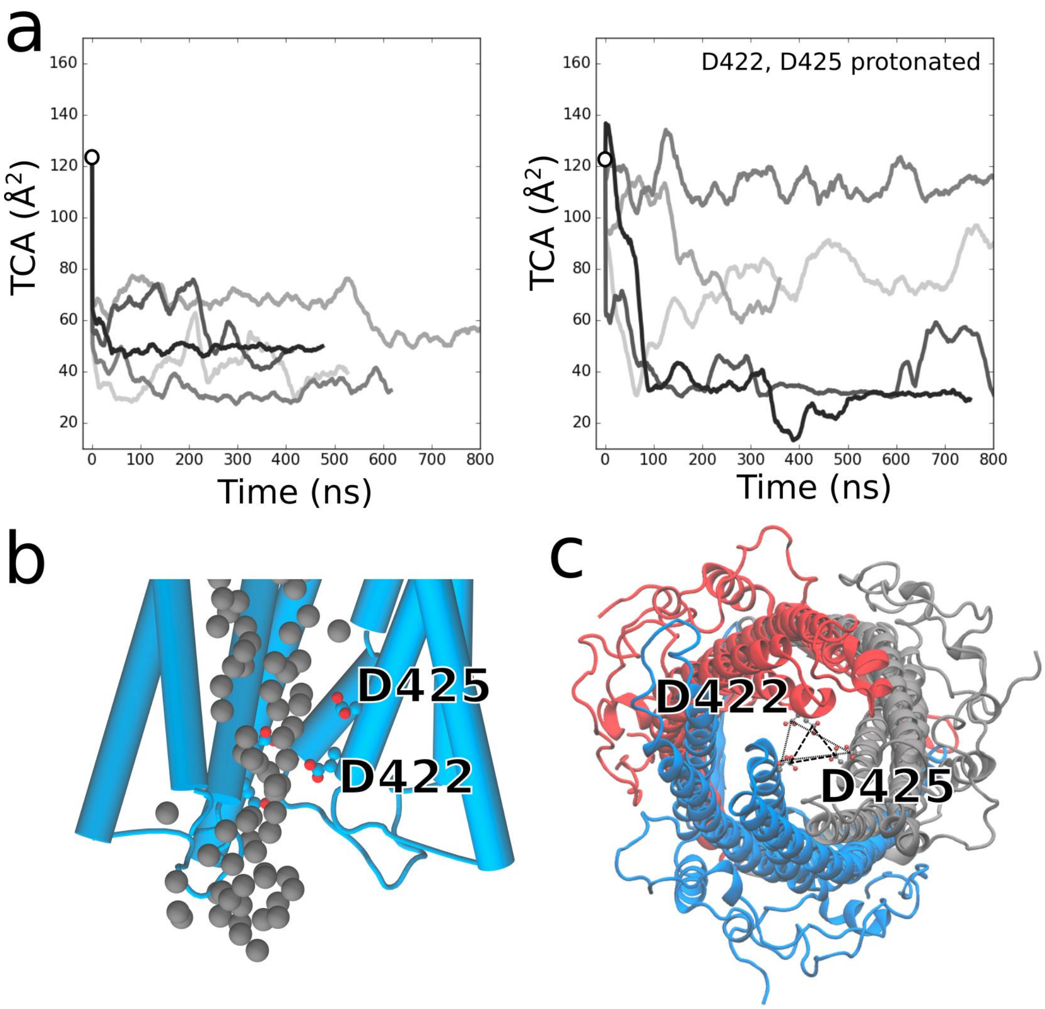

Figure S1. (a) We used the TCA (triangular cross-sectional area) spanned by the Cα atoms of the Asp422 residues in each MtrE monomer to obtain a simple estimate of the cross-sectional area of the periplasmic gate. The black circle indicates the TCA of the crystal structure. In simulations where no acidic residue is in a protonated state, we observe fast closing, with a closed state that remains stable on the time-scale of the simulations (left). In simulations with uncharged protonation aspartate gating rings, we observe an effect on the closing propensity of the channel, due mainly to the disruption of essential intra extra-chain interactions, but a strong tendency to adopt a more closed state is retained in most simulations (right). (b) Representative snapshot of a closed conformation in the gating region with water oxygen atoms in gray. A continuous water wire is consistently observed inside the pore despite its closed state, and dewetting is not observed. (c) Representative closed conformation of MtrE and its TCA.

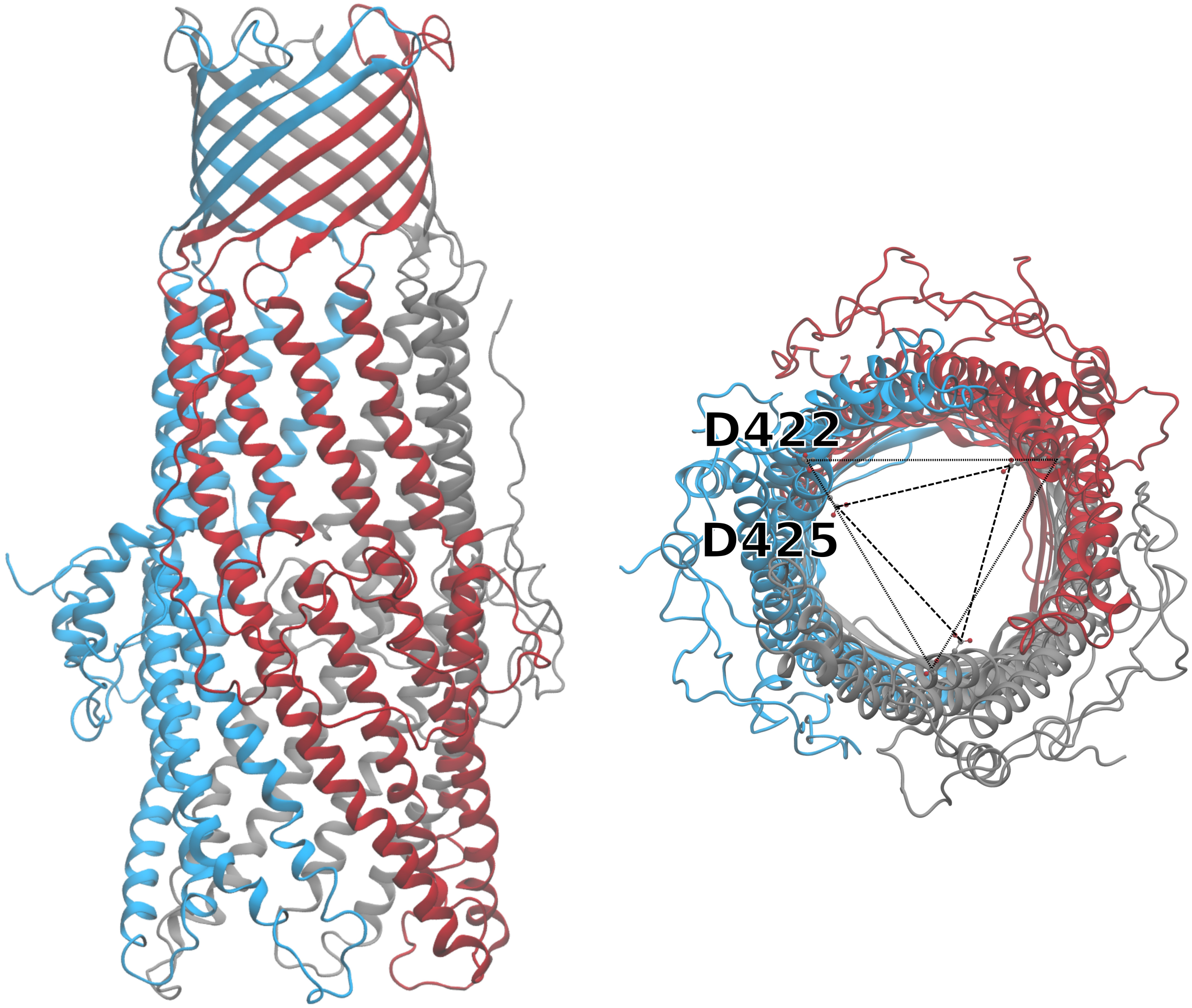

Figure S2. Model of a super-open state of MtrE based on the dilated conformation observed for TolC in the TolC-MacA complex from *E. coli*[28]. In our simulations, we observe a greatly increased conductance of MtrE in this dilated conformation, which is in disagreement with our experimental recordings on the channel.

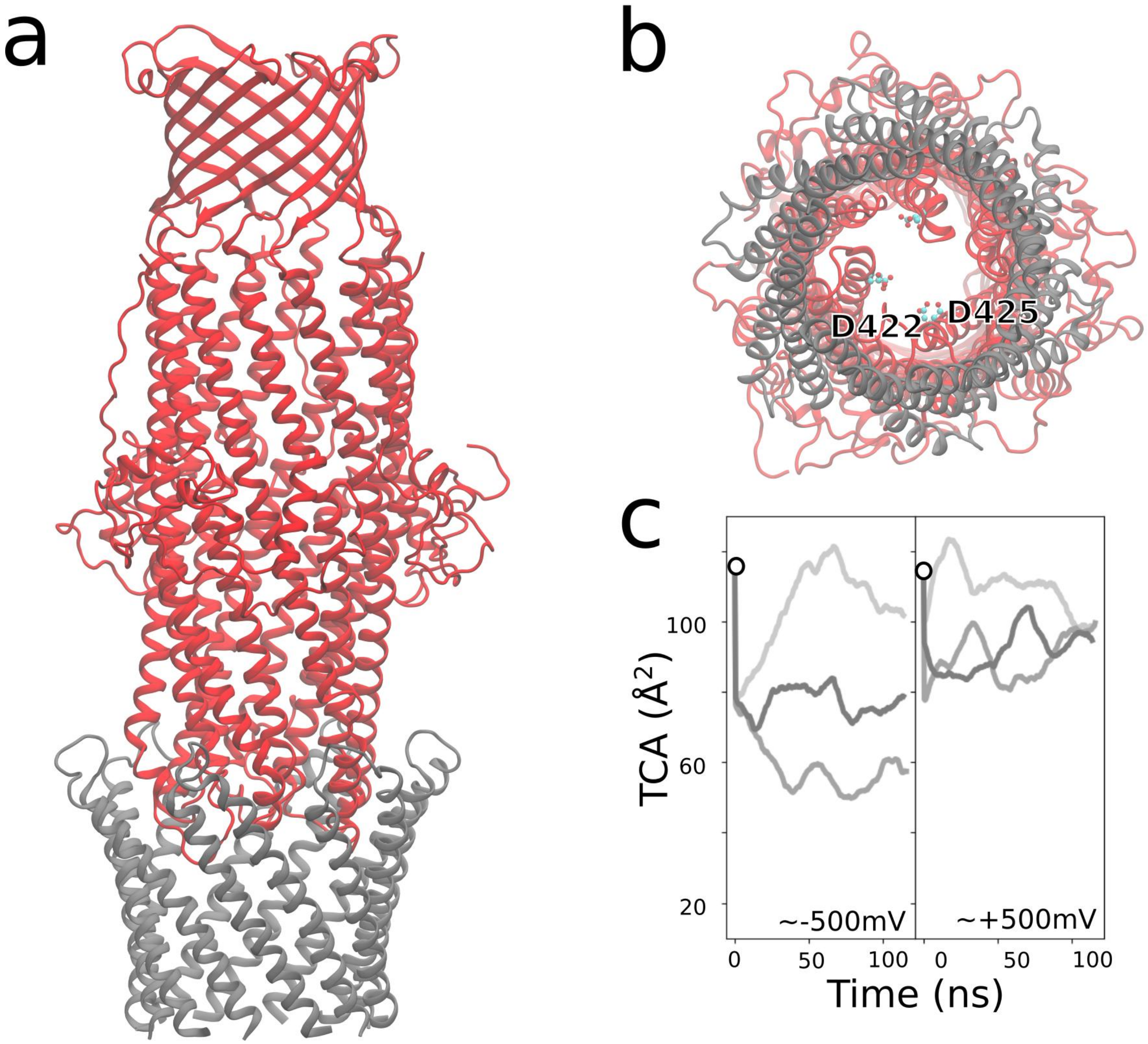

Figure S3. (a-b) Model of a semi-open state of MtrE in complex with the hairpin portion of MtrC, based on a conformation observed in the TolC-AcrAB complex from *E. coli*[8] (a, side view, b, close-up view of the interface region from MtrC). The TCA of this state is similar to the TCA of MtrE in its open-state crystal structure[13], indicated by black circles. (c) TCA of the modeled complex in simulations under voltage.

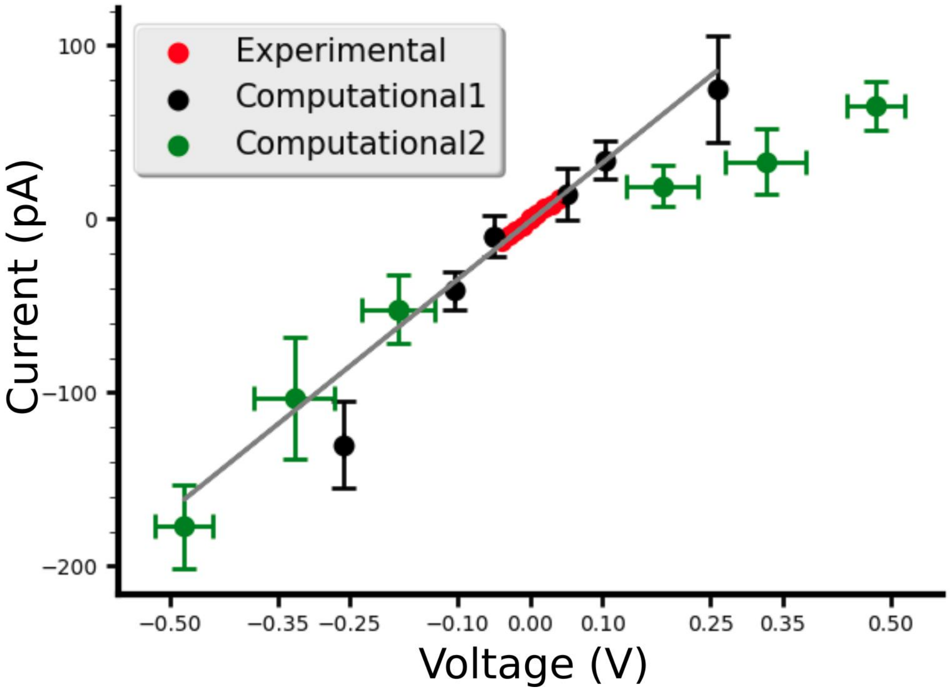

Figure S4. Current-voltage relationship of MtrE from experiment and simulations including voltages far from the experimentally applied ones. Red dots display the experimental current-voltage relationship data in symmetrical 210 mM KCl solution. Black dots show the current-voltage relationship obtained from MD simulations using an external electric field, green dots display data points obtained from CompEL. In the CompEL simulations, voltages of ±0.18, ±0.32 and ±0.48 V were applied by maintaining an ion imbalance of 2, 4, or 6 Cl^- ions, respectively, between two separate compartments. Extrapolating the linear current-voltage relationship obtained in the experimental measurements towards markedly higher voltages (gray line) suggests that at highly positive transmembrane voltages, the data points obtained from an applied electric field offer a better prediction of the MtrE conductance, while by contrast, in the highly negative voltage range, the values from CompEl more closely follow the slope of the extrapolation. Since these voltages are outside of the experimental range, the actual magnitude of the MtrE currents at these potentials remains unclear. Initial analysis shows that the difference arises due to the two distinct ways in which the transmembrane electric field is modelled, and is not related to the force-fields or the membrane lipids used. The emergence of differences between the two approaches is interesting and, since it is out of the scope of the present work focusing on MtrE modulation, will be investigated in the context of a future study.

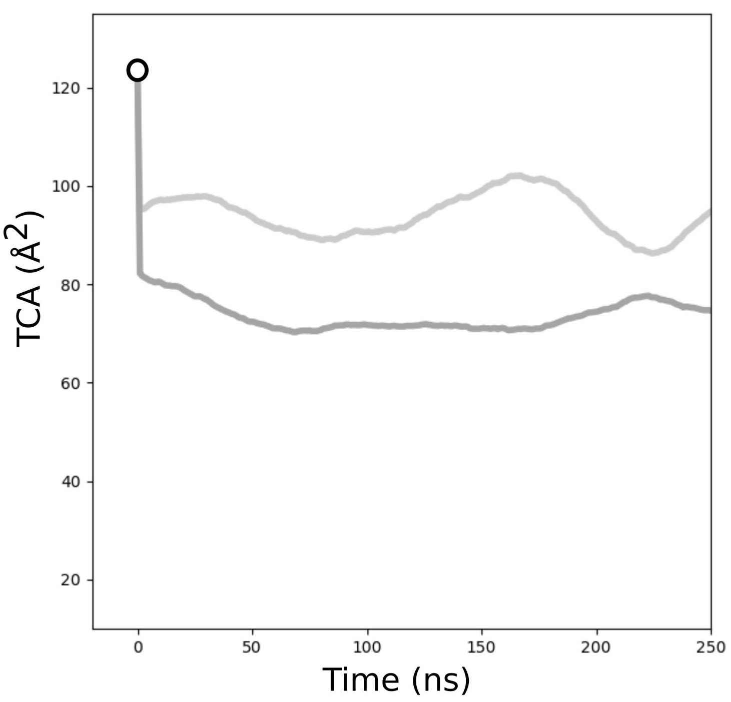

Figure S5. TCA spanned by the Cα atoms of the Asp422 residues in each MtrE monomer from unrestrained simulations of MtrE in a pure POPE bilayer. The area exhibits a rapid transition of the channel to a more closed conformation compared to the crystal structure, which is 125Å^2. This is in line with our observations made in POPC bilayers.

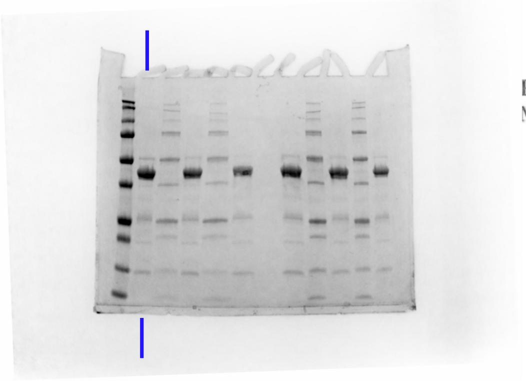

MtrC

Protein sequence coverage: 92%

Matched peptides shown in ***bold red***.

```
  1 MGQPAGREAP APVVGVVTVH PQTVALTVEL PGRLESLRTA DVRAQVGGII
 51 QKRLFQEGSY VRAGQPLYQI DSSTYEAGLE SARAQLATAQ ATLAKADADL
101 ARYKPLVSAD AISKQEYDAA VTAKRSAEAS VKAAQAAIKS AGINLNRSRI
151 TAPISGFIGQ SKVSEGTLLN AGDTTVLATI RQTNPMYVNV TQSASEVMKL
201 RRQIAEGKLL AADGAIAVGI KFDDGTVYPE KGRLLFADPT VDESTGQITL
251 RAAVSNDQNI LMPGLYVRVL MDQVAADNAF IVPQQAVTRG AKDTVMIVNA
301 QGGMEPREVT VAQQQGTNWI VTSGLKDGDK VVVEGISIAG MTGAKKVTPK
351 EWAPSENQAA APQAGVQTAS EAKPASEAKL EHHHHHH
```

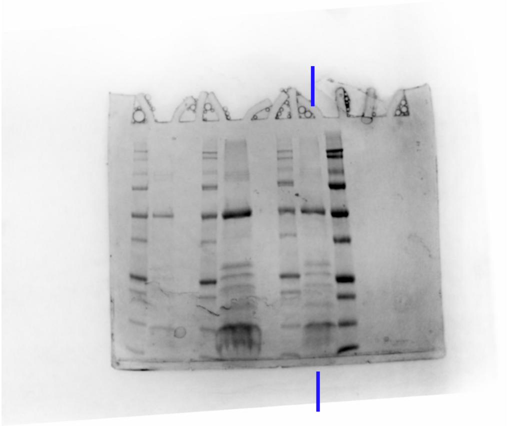

MtrE

Protein sequence coverage: 91%

Matched peptides shown in ***bold red***.

```
  1 MNTTLKTTLT SVAAAFALSA CTMIPQYEQP KVEVAETFQN DTSVSSIRAV
 51 DLGWHDYFAD PRLQKLIDIA LERNTSLRTA VLNSEIYRKQ YMIERNNLLP
101 TLAANANGSR QGSLSGGNVS SSYNVGLGAA SYELDLFGRV RSSSEAALQG
151 YFASVANRDA AHLSLIATVA KAYFNERYAE EAMSLAQRVL KTREETYNAV
201 RIAVQGRRDF RRRPAPAEAL IESAKADYAH AARSREQARN ALATLINRPI
251 PEDLPAGLPL DKQFFVEKLP AGLSSEVLLD RPDIRAAEHA LKQANANIGA
301 ARAAFFPSIR LTGSVGTGSV ELGGLFKSGT GVWAFAPSIT LPIFTWGTNK
351 ANLDVAKLRQ QAQIVAYESA VQSAFQDVAN ALAAREQLDK AYDALSKQSR
401 ASKEALRLVG LRYKHGVSGA LDLLDAERSS YSAEGAALSA QLTRAENLAD
451 LYKALGGGLK RDTQTGKLEH HHHHH
```

Figure S6. SDS PAGE and fingerprint mass-spectrometry (MS) sequence analysis for the MtrC (top) and MtrE (bottom) proteins. The MtrC sample (lane marked blue, top) has been purified to 94.2%, while the membrane protein MtrE (lane marked blue, bottom) is 50.8% pure. Both samples were confirmed as MtrC and MtrE, respectively, by MS identification.
